# The Coexistence of an Intrasellar Adenoma, Lymphocytic Hypophysitis, and Primary Pituitary Lymphoma in a Patient with Acromegaly

**DOI:** 10.1155/2011/941738

**Published:** 2011-10-26

**Authors:** Jose Hernan Martinez, Mariel Davila Martinez, Marcos Mercado de Gorgola, Luis F. Montalvo, Jaime E. Tome

**Affiliations:** ^1^Department of Internal Medicine, San Juan Bautista Medical Center, P.O. Box 4964, Caguas, PR 00726-4964, USA; ^2^Endocrinology Section, San Juan City Hospital, San Juan, PR, USA; ^3^San Juan Bautista School of Medicine, Caguas, PR, USA; ^4^Neurosurgery Department, School of Medicine, University of Puerto Rico, San Juan, PR, USA

## Abstract

The concomitant presence of three histopathologically different entities in the pituitary gland is a rare occurrence. Most publications identify at least two distinct pathologies, mainly, a pituitary adenoma coexisting with a second intrasellar lesion. We present a case of a 71-year-old female referred for evaluation and treatment of acromegaly. Questioning revealed she was experiencing facial palsy, visual disturbances, and syncopal spells for several weeks. When laboratory evaluation showed elevated somatomedin (IGF-I) levels and an oral glucose tolerance test failed to demonstrate any suppression of her growth hormone (GH) values, an MRI of the pituitary revealed a sellar mass. A presumptive diagnosis of pituitary adenoma was established. The patient underwent transsphenoidal resection of the sellar mass, which proved to be a large B-cell lymphoma (Stage I-E) associated with areas of adenoma and lymphocytic hypophysitis.

## 1. Background

Acromegaly is a relatively rare endocrine disease characterized by sustained hypersecretion of growth hormone (GH) from a pituitary adenoma in the majority of cases [1]. Typical features range from subtle signs including thickening of the skin, enlargement of hands, feet, and mandible, and visceromegaly to other florid systemic conditions such as heart failure [2]. GH-dependent factors, such as insulin-like growth factor-I (IGF-I) or somatomedin C, not only have been associated with the classic clinical manifestations of acromegaly but also may be implicated in the increased risk of developing neoplasia [1, 3]. In addition to colon cancer, patients with acromegaly are prone to develop lymphoproliferative cancers such as lymphoma or leukemia [1–3]. The most probable mechanism explaining the above is the close structural homology of these peptides with insulin, therefore stimulating mitogenesis and cellular growth [4]. 

Currently, GH suppression after an oral glucose tolerance test (OGTT) to less than 1 *μ*g/L with sensitive assays and IGF-I levels reduced to the age-gender-adjusted normal range is recommended for acromegaly to be excluded or to be considered nonactive. A mean integrated 24 h level of GH less than 2.5 *μ*g/L also excludes the diagnosis [5, 6]. However, recent studies have argued that the above criteria if applied in atypical acromegalic patients with normal GH-levels, the diagnosis could be missed in up to 25% of patients [7]. But since continuous exposure of the liver and other tissues to even minimally elevated tonic GH levels is sufficient to increase IGF-I production into a supranormal range, IGF-I has been consistently considered as a better reliable indicator for acromegaly and disease activity because it reflects overall GH production [5, 7, 8]. However, alternative tests have been proposed for the diagnosis of acromegaly such as the thyrotropin-releasing hormone (TRH) and Gonadotropin-releasing hormone (GnRH) stimulation tests, where a paradoxical GH increase is seen in acromegalic patients following their administration. Also, abnormal response of GH after L-Dopa administration has been seen in patients with acromegaly [9]. 

The fact that most patients with acromegaly have a GH-secreting pituitary adenoma has emphasized the coexistence between the two and to some extent it has overlooked the possibility of another type of sellar mass as the etiology for acromegaly. Here we describe a patient with coexistent intrasellar adenoma, lymphocytic hypophysitis, and primary pituitary lymphoma (PPL) in association with clinical acromegaly. 

Hypophysitis is a chronic inflammatory process of the pituitary gland presumably of autoimmune etiology. Hypophysitis is classified according to pituitary anatomical site involvement, cause, or histopathological tissue diagnosis. Adenohypophysitis, infundibulohypophysitis, or panhypophysitis have been described depending if the clinical and radiological signs affect the anterior, posterior, the stalk, or both. The cause of hypophysitis has been associated to primary or secondary forms. Primary hypophysitis is the most common and it can manifest as an idiopathic occurrence or as part of a multiorgan disease (polyglandular autoimmune syndrome and IgG related systemic disease). Secondary hypophysitis includes drug-induced agents like interferon alpha administration or cases where hypophysitis is part of a multiorgan systemic involvement (tuberculosis, sarcoidosis, wegener granulomatosis) or inflammation of the pituitary gland due to the presence of another sellar lesion (pituitary adenoma, craniopharyngioma, Rathke cleft cyst). From the histopathological point of view two distinct lesions are found, lymphocytic and granulomatous hypophysitis, and four variants, mainly, xanthomatous, necrotizing, plasma cell rich, and IgG4-related hypophysitis. Lymphocytic hypophysitis is the most common, occurring uniquely in relation to pregnancy or the postpartum period in about 40% of the women [10, 11]. PPL remains an exceedingly rare entity in immunocompetent individuals [12–14]. The diagnosis once anecdotically reported has increased in frequency due to progressive refinement of endocrine tests and imaging procedures. Symptoms of PPL are usually associated with headaches, cranial nerve involvement, visual field defects, and hypopituitarism. Among the risk factors for PPL, besides AIDS, are pituitary adenoma and lymphocytic hypophysitis [15, 16]. To our knowledge, this is the first case presenting the coexistence between the three pathological entities and acromegaly in the medical literature. 

## 2. Case Report

A 71-year-old female was referred to our endocrinology clinic on March 2009, for evaluation and treatment of acromegaly. Her past medical history included arrhythmia, Hashimoto's thyroiditis, and previous modified radical mastectomy for which she did not received adjuvant therapy. She was HIV-negative with no past history of immunosuppresive therapy. When the patient was initially examined typical and obvious acromegalic features were evident. Questioning revealed she was experiencing facial palsy, visual disturbances, and syncopal spells for several weeks. Although she was under a tightly monitored regimen for her medical conditions, a complete work-up was established. The patient underwent extensive laboratory evaluations, which were unrevealing. Basal hormonal studies showed values not indicative of hypopituitarism. Baseline serum prolactin of 15.17 ng/mL (normal range, <16.5), total thyroxine of 6.8 *μ*g/dL (normal range, 4.5 to 12.5), thyroid-stimulating hormone of 5.4 *μ*U/mL (normal range, 0.45 to 4.5), ACTH of 24 pg/mL, and a morning serum cortisol of 11.8 *μ*g/dL (normal range, 5.0 to 25.0). Signs or symptoms of posterior pituitary involvement were absent. Notable findings were raised somatomedin (IGF-I) levels and an oral glucose tolerance test that failed to demonstrate any suppression of her GH levels (Table 1). Radiographic evidence of head and hands showed widening of the cranial vault diploe space mainly at the frontal region and “sausage-like” digits and tufting of the terminal phalanx suggesting the clinical diagnosis of acromegaly. 

**Figure 1:**
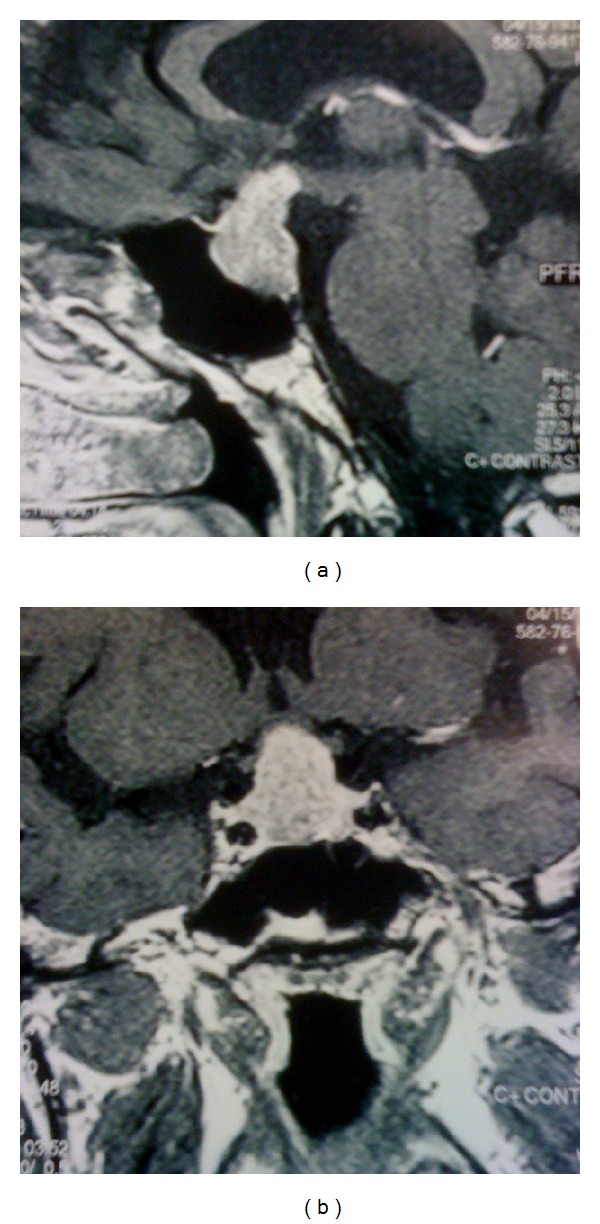
MRI T1-weighted scans on admission: (a) sagittal section, (b) coronal section.

Neurological examination was compatible with a bilateral lesion of the optic nerve (bilateral temporal hemianopsia), further confirmed by visual field examination and evoked visual potentials. MRI of the pituitary revealed a 2.3 × 2.2 × 1.7 cm well-circumscribed mass extending into the suprasellar cistern and compressing the optic chiasm. There was also penetration into the right cavernous sinus with encasement of the right cavernous carotid artery. After gadolinium administration there was nonhomogenous enhancement of the tumor, with no intradural invasion noted. No other brain lesions were seen (Figures 1(a)-1(b)). A presumptive diagnosis of pituitary adenoma was made. 

**Figure 2:**
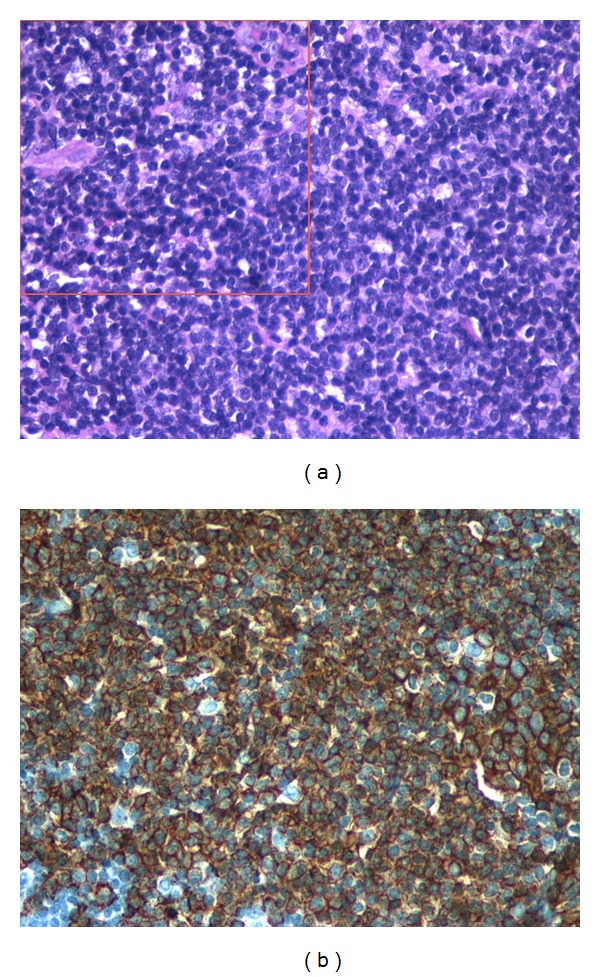
(a) Tissue of pituitary gland with cell-rich infiltrate of lymphoma. Large cells with nuclei display prominent nucleoli (magnification 40x). On red square adjacent area of lymphocytic hypophysitis. (b) Tumor cells express the B-cell marker CD20 and show dark membrane staining (magnification 40x).

**Figure 3:**
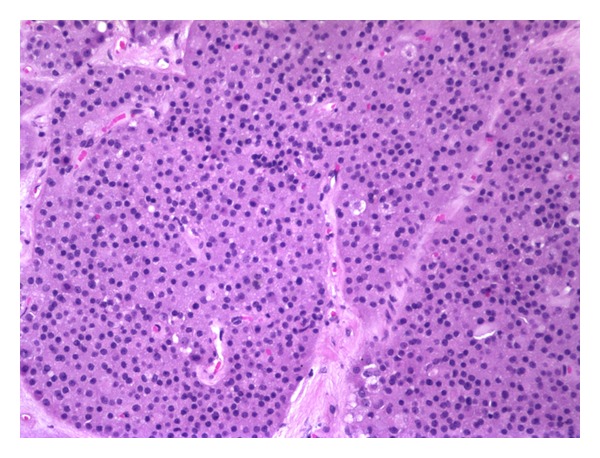
Histopathological finding of the adenomatous area with its typical monotonous cells (magnification 20x).

The patient underwent transsphenoidal resection of the pituitary mass (March 26, 2009) without any intraoperative or postsurgical complications. At light microscopy, the mass contained loosely cohesive cells with numerous mitotic figures. A group of cells suggestive of lymphocytic hypophysitis were also identified adjacent to the tumor (Figure 2(a)). Moreover, the tumor showed positive immunostain for B-cell markers CD20 and CD79 alpha (Figure 2(b)). Positive Ki67 marker suggested active proliferation in the tumor area and positive Bcl6 marker confirmed the germinal center origin of the malignant lymphocytes (Table 2). In addition, a nonfunctional adenomatous area with its typical features was recognized close to the lymphoma (Figure 3).

**Figure 4:**
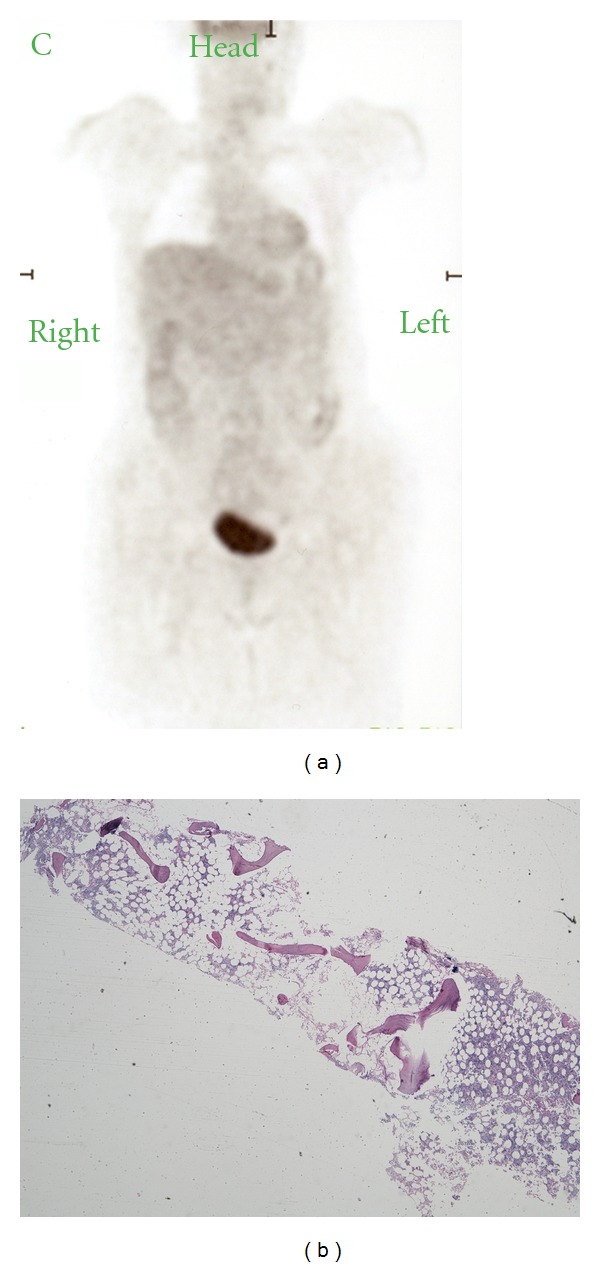
(a) Negative whole body PET/CT scan. (b) Bone marrow aspiration sample evidencing normal parenchyma.

After surgery, the patient received a 5040cGy course of intensity-modulated radiation delivered to the pituitary, sphenoid sinus, posterior nasal cavity, nasopharynx, and oropharynx over 6 weeks in 180-cGy fractions. Radiation therapy was complicated by oropharyngeal thrush which required interruption of treatment for one week. Since the lymphoma was very well localized (Stage I-E) and whole body 18F-FDG PET/CT and bone marrow biopsy excluded disseminated disease, no systemic chemotherapy was suggested (Figure 4). One year after radiotherapy the patient has remained completely asymptomatic. Results of inflammatory markers IL-1, IL-6 and IGFBP-3 after surgery and radiation therapy were within normal limits confirming the nonclinical activity and remission of acromegaly and lymphoma.

## 3. Discussion

Our patient was found to have three histopathologically different entities coexisting in the sellar region; a non-functional adenoma, lymphocytic hypophysitis, and a primary pituitary lymphoma. A preoperative presumptive diagnosis of only a pituitary adenoma was made on the basis of the patient's presentation, imaging findings, and the extensive research data pointing, that is, the most common cause of a mass in the sella (up to 10%–15%) [15, 17]. Hence, other nonpituitary sellar masses should be considered: cell rest tumors, germ cell tumors, gliomas, meningiomas, metastatic tumors, vascular lesions, granulomatous, infectious, and inflammatory processes, and even lymphomas [17].

Primary CNS lymphoma (PCNSL) is now thought to constitute 3% of all intracranial neoplasms [13, 15]. However, the incidence of PCNSL has been increasing in both immunocompetent and immunocompromised individuals. The vast majority are large B-cell lymphomas, as in our case [12]. Most are periventricular in location typically involving the corpus callosum, basal ganglia, cerebellum, orbits, and cranial nerves; involvement of the pituitary is rare [12, 13, 16]. More than 50% of patients with PCNSL, especially PPL, show signs of hypopituitarism. Other characteristic clinical findings of pituitary PCNSL are usually visual compromise, cranial nerve abnormalities, and nonspecific symptoms such as headaches. Modest hyperprolactinemia secondary to hypothalamic-pituitary stalk compression is common. In none of the cases reported in some detail in the literature about PPL, acromegaly is part of the clinical manifestation [12–16, 18, 19]. In our case, it was the initial clinical manifestation.

The origin of PPL still remains unknown. Some etiologic theories suggest that the tumor may arise from resident lymphoid tissue in the CNS, while others consider the possibility of normal lymphocytes migrating to the sellar region due to inflammatory processes and subsequently undergoing malignant transformation [3, 13]. This malignant transformation could be attributed to the presence of GH, IGF-I, IGF-II, and insulin receptors as they have been recognized on normal lymphocytes and on human lymphoblasts [3, 4, 20]. In our case the idea of migrating lymphocytes with further malignant transformation should be highly entertained due to the patient's medical history of Hashimoto's thyroiditis and breast cancer (both with an inflammatory component). As to further emphasize the importance of the inflammatory component, lymphocytic hypophysitis and pituitary adenoma have been both associated with the eventual development of PPL [15, 21]. 

Based on the obvious clinical features, the elevated serum IGF-I levels and nonsuppressible GH levels after OGTT, it is evident that our patient has acromegaly. But from where is the GH actually coming from? Our patient had no evidence of systemic diseases. Hence, a secondary GH-releasing tumor is unlikely. Since in our patient immunostains of the adenomatous component was negative for all anterior pituitary hormones, one plausible explanation is the role of the tumor itself in the basal production of GH-dependent factors, thus contributing to the neoplastic transformation of cells and the clinical manifestation of acromegaly. Immunoreactivity has established the production of GH and IGF-I by lymphoblasts and lymphocytes [3, 22]. In addition, the recognition of somatostatin receptors on malignant lymphomas has been reported [2, 23]. Data on these studies suggest that the absence of the expression of somatostatin receptors may take part on the GH secretion because of the lack of inhibitory effects of endogenous somatostatin. Moreover, clinical cases presenting both lymphocytic hypophysitis and elevated IGF-I levels have also been reported, further emphasizing the role of the inflammatory cells themselves in the production of GH and IGF-I [24, 25]. 

The concomitant presence of a pituitary adenoma with other morphologically different lesions is an uncommon phenomenon [26]. So in our opinion this clinical case has four major outstanding features: (1) the unusual coexistence of lymphocytic hypophysitis and adenomatous tumor growth as possible risk factors for PPL, (2) the hypersecretion of GH and IGF-I whose point of origin is not altogether clear, (3) the occurrence of lymphocytic hypophysitis in a postmenopausal woman, and (4) that the majority of patients with PPL are male. 

## 4. Conclusions

Although the case we described here has definite clinical, radiological, and biochemical evidence to support the diagnosis of acromegaly, the source of GH is not completely understood. However, possible theories might be raised including the slow, continuous hypersecretion of GH from the adenoma or even from the lymphocytes of the lymphoma as previously explained. Future lines of research should be directed to the understanding of the pathological and immunological mechanisms that lead to a better characterization of such processes. We also propose that although acromegaly and pituitary adenoma are highly intertwined across the medical literature, careful exclusion of primary neoplasm and other inflammatory lesions of the pituitary gland should be warranted.

## Figures and Tables

**Table 1:** Acromegaly diagnosis and disease activity.

Parameter	At Diagnosis	One-year followup
Random GH	1.32 *μ*g/L	0.55 *μ*g/L
(<2.5 *μ*g/L)		
2 hrs post-glucose load	1.12 *μ*g/L	0.48 *μ*g/L
(<1 *μ*g/L)		
5 hrs post-glucose load	1.57 *μ*g/L	0.87 *μ*g/L
(<1 *μ*g/L)		
IGF-I	221.0 ng/mL	182.0 ng/mL
(64–188 ng/mL)		
IGFBP-3	—	4.00 mg/L
(2.8–5.7 mg/L)		
IL-1	—	0.40 pg/mL
(0–3.9 pg/mL)		
IL-6	—	<2.00 pg/mL
(0–14.0 pg/mL)		

**Table 2:** Immunostainings of adenoma cells and lymphoma.

Marker	Adenoma	B-cell lymphoma
GH	−	
Prolactin	−	
ACTH	−	
TSH	−	
Ki67		90% of cells
Bcl2		−
Bcl6		+
CD20		+
CD79a		+
CD38		−
CD44		+
CD3		+
CD5		+
CD10		+
MUM1		−

− = negative, + = positive.
